# Associations between N-Terminal Pro-B-Type Natriuretic Peptide, Body Fluid Imbalance and Quality of Life in Patients Undergoing Hemodialysis: A Cross-Sectional Study

**DOI:** 10.3390/jcm12237356

**Published:** 2023-11-28

**Authors:** Keisuke Yamazaki, Shingo Ishii, Mai Hitaka, Motoyuki Masai, Yasushi Ohashi

**Affiliations:** 1 Department of Nephrology, Toho University Sakura Medical Center, Chiba 285-8741, Japan; keisuke.yamazaki@med.toho-u.ac.jp (K.Y.); shingo.ishii@med.toho-u.ac.jp (S.I.); mai.hitaka@med.toho-u.ac.jp (M.H.); 2 Department of Urology, Mihama Hospital, Chiba 261-0013, Japan; m.masai@seijinkai.org

**Keywords:** geriatric nutritional risk index, hemodialysis, inflammation, malnutrition, natriuretic peptides, phase angle, quality of life, sarcopenia, skeletal muscle mass index, volume overload

## Abstract

Natriuretic peptides may be associated with the complex interaction between malnutrition and fluid overload. This study assessed the relationship between N-terminal pro-B-type natriuretic peptide (NT-proBNP), body fluid composition, and quality of life (QOL) domains. A multicenter, cross-sectional study was conducted between 2019 and 2022. The QOL survey of 322 patients undergoing maintenance hemodialysis (227 men and 95 women; mean age, 65 ± 12 years) was conducted using the Kidney Disease QOL-Short Form v. 1.3. The patients in the higher NT-proBNP quartile group were older and had a longer dialysis vintage; lower body mass index, serum albumin, blood urea nitrogen, creatinine, sodium, uric acid, total cholesterol, triglycerides, and hemoglobin levels; lower geriatric nutritional risk index (GNRI), skeretal mascle mass index, and phase angle (PhA); and higher pre- and post-dialysis systolic blood pressure (BP), cardiothoracic index, and C-reactive protein (CRP) (*p* < 0.05). Multivariate analysis revealed that post-dialysis systolic BP, CRP, and GNRI or PhA were independently associated with NT-proBNP. The higher NT-proBNP group experienced muscle attenuation and/or inflammation and an enlarged left atrium. Consequently, the elevated NT-proBNP by such an imbalance in body fluid composition is associated with lower health-related QOL.

## 1. Introduction

Cardiovascular diseases are the most common cause of death in patients undergoing hemodialysis [1]. The progression of chronic kidney disease stage leads to gradual sodium retention and extracellular fluid expansion, resulting in the release of compensatory natriuretic peptide in response to increased left ventricular wall stress and stretching. Previous studies have identified N-terminal pro-B-type natriuretic peptide (NT-proBNP) as a predictive factor of cardiac events and mortality in hemodialysis [2,3,4,5]. However, NT-proBNP is multilaterally correlated with inflammation and protein energy wasting syndrome (PEW) as well as volume overload. [6,7,8]. The clinical significance of NT-proBNP for volume overload has not been fully established in patients undergoing hemodialysis [9,10]. In fact, the natriuretic peptide was associated with 2-year mortality in a large hemodialysis cohort even with or without cardiovascular disease [11].

Patients undergoing hemodialysis have a significantly reduced quality of life (QOL) compared with the general population [12,13,14]. However, clinical indicators of the QOL of patients undergoing hemodialysis have not yet been identified. It has been reported that the post-dialysis recovery time is strongly correlated with QOL scores. The prevalence of post-dialysis fatigue ranges from 60% to 97% [15]. Several factors including aging, malnutrition, anemia, inflammatory state, inadequate dialysis, and the ultrafiltration rate (UFR) play a role in the pathogenesis of post-dialysis fatigue [16].

In the current aging Japanese society, malnutrition and sarcopenia in patients undergoing hemodialysis are widely recognized as indicators of poor health that can decrease the QOL of older people [17]. We hypothesized that natriuretic peptide, which is associated with malnutrition, inflammation, and volume overload, may be an effective indicator of low QOL. We investigated the associations between NT-proBNP, body fluid imbalance, and QOL in patients undergoing hemodialysis.

## 2. Materials and Methods

### 2.1. Study Design and Participants

This is a sub-analysis study of patients who consented to a QOL questionnaire in our previous cross-sectional study. The detailed study design has been published elsewhere [18]. Participants were recruited from four maintenance hemodialysis clinics (Seijinkai Mihama Hospital, Seijinkai Mihama Sakura Clinic, Seijinkai Narita Clinic, and Seijinkai Katori Clinic) between 2019 and 2022. During this period, a total of 1094 patients (777 men and 317 women) underwent hemodialysis at these centers. We reviewed the electronic medical records and excluded patients if they met any of the following criteria: underwent coronary and/or valvular intervention or suffered a myocardial infarction within the last 6 months; had been hospitalized for an unscheduled dialysis for heart failure treatment in the last 6 months; had echocardiographic evidence of a left ventricular ejection fraction (LVEF) of <40%; had a contraindication to bioimpedance measurements, such as a pacemaker, joint replacement, or mechanical heart valve; were pregnant; or had major amputations, advanced malignancy, or dementia. Consequently, patients (aged ≥ 20 years) who were undergoing maintenance dialysis for at least ≥90 days and had a stable dialysis prescription for 30 days at the time of recruitment were considered eligible. Finally, 322 patients who provided informed consent to the QOL questionnaire were included in this study.

This study was approved by the Ethics Committee of Toho University Sakura Medical Center, Tokyo, Japan (Approval no. S21073|26 April 2022 [S18086|27 December 2018]), and was conducted in accordance with the Declaration of Helsinki. Informed consent was obtained from all participants.

### 2.2. Data Collection

Patient’s age, sex, diabetes mellitus status, hemodialysis vintage, comorbidity of cardiovascular disease, height, body weight, and pre- and post-dialysis blood pressure (BP) were recorded as baseline data. The following standard laboratory parameters were measured during the long-interval hemodialysis session (i.e., the 1st time of dialysis session of week) at the beginning of the month: total protein, serum albumin, blood urea nitrogen (BUN), serum creatinine (Cr), sodium, potassium, chloride, calcium, phosphorus, uric acid, total cholesterol (TC), triglyceride (TG), glucose, Fe, total iron-binding capacity, ferritin, hemoglobin (Hb), hematocrit, and C-reactive protein (CRP). Dialysis adequacy, which refers to the urea reduction ratio and single-pool Kt/Vurea, was measured using the Shinzato formula [19]. The geriatric nutritional risk index (GNRI) was calculated as using the following formula: 14.89 + serum albumin (g/dL) + (41.7 × body weight/ideal body weight). The ideal body weight was determined based on patient’s height, and 22 kg/m^2^ was considered as the ideal body mass index (BMI) [20]. Pre-dialysis NT-proBNP levels were measured using an electrochemiluminescence immunoassay system (Cobas8000 e801 module; Roche Diagnostics K.K., Tokyo, Japan). Pre-dialysis chest X-ray images were obtained to evaluate the cardiothoracic ratio (CTR), which can help in assessing volume overload at the peak weight gain. CTR was calculated as the ratio of the maximum transverse cardiac diameter (mm) to the maximum thoracic diameter (mm). As a routine clinical practice, annual transthoracic echocardiographic examinations were performed by a single experienced cardiologist (median (10th–90th percentile) duration between body fluid composition analysis and echocardiographic examinations was −70 days (−268 to 68 days)). The left ventricular mass (LVM) was calculated using the Devereux equation [21], and the LVM index (LVMI) was calculated as the ratio of the LVM to the body surface area.

### 2.3. Assessment of Body Fluid Composition

Standard multi-frequency-bioimpedance analysis (MF-BIA) was performed with the patient in the supine position placed on a bed after hemodialysis. For body composition measurements, a segmental MF-BIA device (Inbody S10®; InBody Co., Ltd., Seoul, Republic of Korea; https://inbodyusa.com/ accessed on 1 November 2019) was used. Microprocessor-controlled switches and an impedance analyzer were activated, and segmental resistances of the arms, trunk, and legs were measured at four frequencies (5, 50, 250, and 500 kHz). Subsequently, total body water, intracellular water (ICW), extracellular water (ECW), ECW to ICW ratio, protein, mineral, fat, fat-free mass, and body cell mass were calculated using the InBody S10^®^ software. The phase angle (PhA) was calculated based on the resistance (R) and reactance (Xc; measured at 50 kHz) using the following equation: PhA (°) = arctangent (Xc/R) × (180/π) [22]. The skeletal muscle mass index (SMI) (appendicular skeletal muscle mass/height^2^, kg/m^2^) was measured as the sum of lean soft tissue of the two upper and lower limbs.

### 2.4. QOL Assessment

Participants completed the Kidney Disease QOL-Short Form (KDQOL-SF) v. 1.3 survey, which is a modified version of SF-36 that was developed specifically for patients undergoing dialysis, within the first 60 min of their dialysis treatment [23]. The used form was validated for Japanese patients [24]. The kidney disease-specific questions comprised ten evaluation items: symptoms, effects of kidney disease on daily life, burden of kidney disease, work status, cognitive function, quality of social interaction, sleep, social support, dialysis staff encouragement, and patient satisfaction. In addition, factors in the SF-36 health survey including physical functioning, role physical, bodily pain, general health, vitality, social functioning, role emotional, and emotional well-being can be evaluated.

### 2.5. Statistical Analyses

The measured values were expressed as medians (interquartile ranges). Statistical significance was assessed using a linear regression model for continuous variables and Pearson’s chi-square test for categorical variables. Correlations between the variables were determined using the Pearson product–moment correlation coefficient. To evaluate independent associations, explanatory variables that showed a significant relationship (*p* < 0.05) with NT-proBNP were analyzed using multivariate analysis. However, BUN, Cr, UA, TC, TG, and sodium were excluded from the variables because they were considered confounders of NT-proBNP, GNRI, or PhA. In addition, age, BMI, and serum albumin were also excluded from the variables in Model 1 because they are included in the formula for GNRI; then, GNRI was excluded from variables in Model 2 because it was considered to have multicollinearity PhA. To evaluate the independent associations of NT-proBNP for QOL domains, explanatory variables that showed a significant relationship (*p* < 0.05) with each QOL domain were analyzed using multivariate analysis. Data were analyzed using JMP pro (version 17.0; SAS Institute, Inc., Cary, NC, USA). *p* of <0.05 was considered statistically significant.

## 3. Results

### 3.1. Population Characteristics

The characteristics of included patients (227 men and 95 women; mean age, 65 ± 12 years) are presented based on NT-proBNP quartiles in Table 1. The quartile values were 1715, 3420, and 7178 pg/mL. The higher NT-proBNP quartile group was older and had a longer dialysis vintage; lower BMI, serum albumin, BUN, Cr, sodium, UA, TC, TG, and Hb levels; lower GNRI; and higher pre- and post-dialysis systolic BP, CTR, and CRP (*p* < 0.05). In the multivariate analysis, post-dialysis systolic BP, CRP, and GNRI were independently associated with NT-proBNP in model 1, or post-dialysis systolic BP, CRP, and PhA remained independently associated with NT-proBNP in model 2 (Table 2).

### 3.2. Association between Body Fluid Imbalance and NT-proBNP

**Figure 1:**
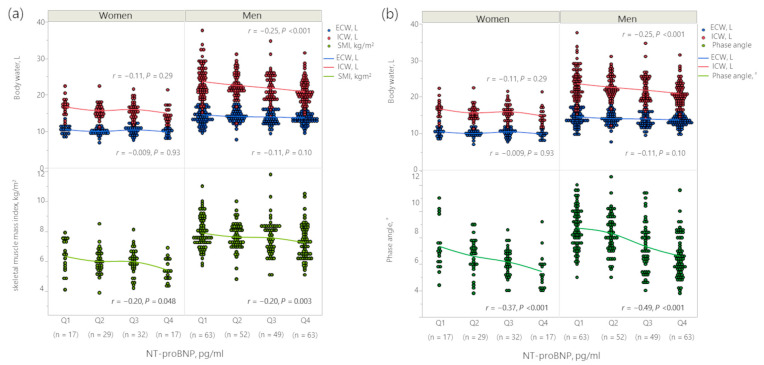
(**a**) Body fluid composition with skeletal muscle mass index based on the NT-proBNP quartiles and (**b**) body fluid composition with phase angle based on the NT-proBNP quartiles.

The body fluid composition of men and women according to the log_10_-transformed NT-proBNP level quartiles are shown in Figure 1. The log_10_-transformed NT-proBNP had a weak negative correlation with ICW content in men (*r* = −0.25, *p* < 0.001); however, the correlation was insignificant in women (*r* = −0.11, *p* = 0.29). Nevertheless, the log_10_-transformed NT-proBNP had no significant correlation with ECW content in both men and women (men: *r* = −0.11, *p* = 0.10; women: *r* = −0.009, *p* = 0.93). Interestingly, the log_10_-transformed NT-proBNP had a significant negative correlation with SMI (men: *r* = −0.20, *p* = 0.003; women: *r* = −0.20, *p* = 0.048) (Figure 1a) and PhAs (men: *r* = −0.37, *p* < 0.001; women: *r* = −0.49, *p* < 0.001) (Figure 1b). Detailed data are shown in Supplementary Tables S1 and S2.

**Figure 2:**
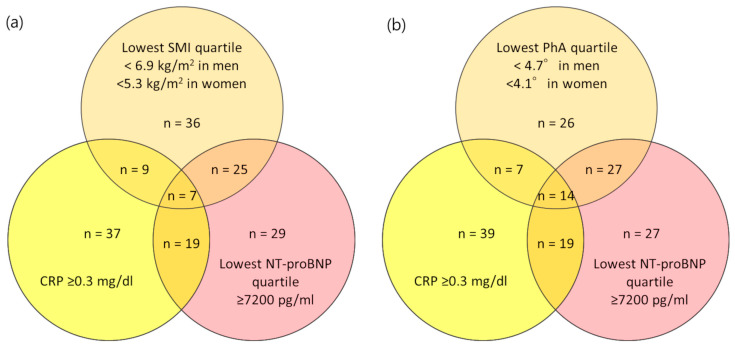
Interactions between (**a**) NT-proBNP, skeletal muscle mass index, and inflammation and (**b**) NT-proBNP, phase angle, and inflammation as part of a multidimensional spectrum.

In this study, 51 of the 80 patients (63.8%) with the highest quartile of NT-proBNP (≥7200 pg/mL) had the lowest SMI quartile (6.9 kg/m^2^ in men and 5.3 kg/m^2^ in women) and/or inflammation (CRP ≥0.3 mg/dL); moreover, 53 of these (66.3%) had the lowest PhA quartile (4.7° in men and 4.1° in women) and/or inflammation (Figure 2).

### 3.3. Association between NT-proBNP and Echocardiographic Findings

Echocardiography findings performed within 1 year are shown in Table 3 according to the NT-proBNP quartiles. The higher NT-proBNP quartile group had a wide left atrial dimension, left ventricular end-diastolic diameter, and left ventricular end-systolic diameter; thick left ventricular posterior wall thickness and interventricular septum thickness; low EF; and heavy LVMI (*p* < 0.05).

### 3.4. Association between NT-proBNP and QOL

**Figure 3:**
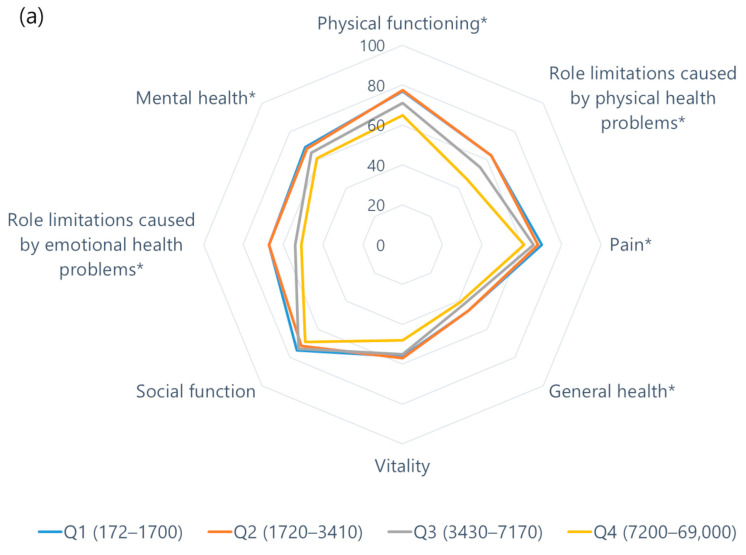

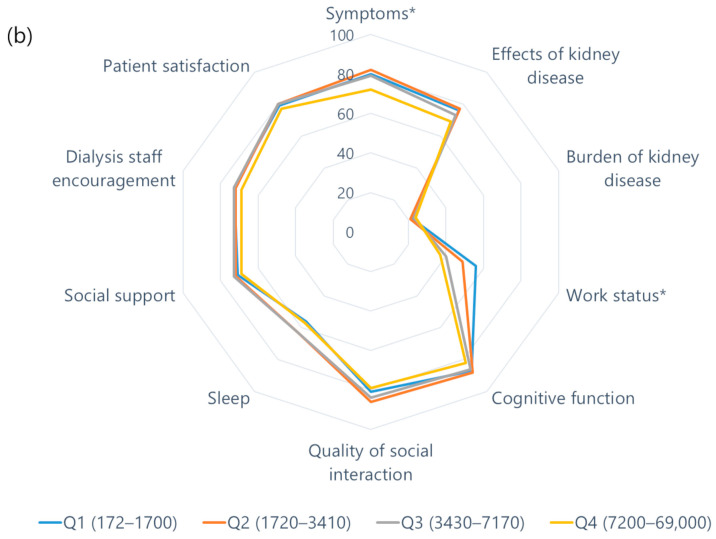
(**a**) Health-related QOL domains by NT-proBNP quartiles and (**b**) kidney disease-specific domains by NT-proBNP quartiles. * *p* < 0.05, Correlation between NT-proBNP and QOL domains.

The mean values of QOL domains according to the NT-proBNP level quartiles are shown in Table 4. Overall, the scores of the health-related QOL domains and kidney disease-specific domains appeared to be low in this study population, particularly on role limitations caused by physical health problems, general health, vitality, role limitations because of emotional health problems, burden of kidney disease, work status, and sleep (Figure 3). Among them, the higher NT-proBNP quartile group had significantly lower generic SF-36 scores. Conversely, a significant difference was observed only in symptoms and work status in the kidney disease-specific domains. In the multivariate analysis, NT-proBNP remained independently associated with each health-related QOL domains of physical functioning, role limitations owing to physical health problems, bodily pain, general health, role limitations because of emotional health problems, emotional well-being and with each kidney disease-specific domain of symptoms and work status (Supplementary Table S3).

## 4. Discussion

This study revealed that NT-proBNP in patients undergoing hemodialysis was significantly associated with BP, CRP, and GNRI or PhA. Most patients in the higher NT-proBNP quartiles had muscle attenuation and/or inflammation, enlarged left atrium by volume overload, and hypertension. The elevated NT-proBNP by such imbalance in body fluid composition was associated with lower health-related QOL.

Natriuretic peptides are neurohormones synthesized in cardiac myocytes in response to increased left ventricular wall stress and stretching, which are reportedly associated with fluid volume accumulation [9,25]. Previous studies on NT-proBNP revealed its potential as a highly predictive biomarker for subsequent cardiac events and mortality in patients undergoing hemodialysis [2,3,4,5,26,27]. In this study, NT-proBNP was correlated with LAD, EF, and LVMI. However, whether NT-proBNP is a marker of volume overload or a marker of left ventricular dysfunction remains unclear. A review article showed that NT-proBNP was correlated with PEW syndrome and inflammation in patients undergoing hemodialysis [28]. In fact, 63.8% of patients with NT-proBNP ≥7200 pg/mL had low SMI and/or inflammation, and 53 patients (66.3%) had low PhA and/or inflammation. The relationship between NT-proBNP and PEW may be attributed to the following reasons: (1) PEW directly affects NT-proBNP by influencing ventricular remodeling; (2) complex interactions among malnutrition, inflammation, and fluid overload; and (3) significant involvement of adipose tissues. NT-proBNP exhibits a lipolytic effect on adipose tissue and enhances the oxidative capacity of human skeletal muscles. A connection with the heart adipose tissue and link between NT-proBNP, PEW, and the appetite-modulating hormone ghrelin have been suggested [29]. In addition, we speculate that patients undergoing hemodialysis may be more prone to malnutrition–inflammation–fluid overload complex (MIFO) syndrome. Apoptotic body fluid imbalance due to aging and PEW is less likely to preserve excess fluid because the cell area is replaced by the interstitium in lean tissue. Furthermore, adipose tissues may function as a buffer for fluid accumulation because they expand the extracellular fluid area [30]; for example, we often encounter patients who are obese and have no symptoms, even with fluid accumulation of 6.0–7.0 L.

Most older patients undergoing hemodialysis have limited life expectancy, multiple comorbidities, and functional impairment. Fatigue is associated with poor health-related QOL and is an important predictor of survival in patients undergoing hemodialysis. However, its pathophysiology is multifactorial, which may include decreased oxygen supply and increased reliance on anaerobic metabolism to cause lactic acidosis in response to exertion, chronic metabolic acidosis, hyperphosphatemia in skeletal muscle myocytes, PEW, sarcopenia, and depression [31]. We suspect that these fatigue-causing mechanisms also elevate natriuretic peptide levels. Therefore, we believe that NT-proBNP is strongly associated with health-related QOL. Most of the study population, particularly patients with higher NT-proBNP levels, felt they had a limited daily life functioning while undergoing maintenance hemodialysis. Currently, few reports have described an association between fatigue and natriuretic peptide in dialysis patients [32].

This study has several limitations. First, this four-center study included 322 of the 1094 patients (29.4%) who were undergoing maintenance hemodialysis in clinics; hence, a generalization of our results to the general population may not be valid. Second, we could not quantitatively measure overhydration with a body cell mass device. Therefore, we determined the presence or absence of volume overload based on the elevated NT-proBNP. Third, we could not quantitatively measure overhydration using a body cell mass device. Fourth, performing blood tests for measuring NT-proBNP and body fluid composition analysis are deemed unnecessary on the same day of hemodialysis (a median (10th–90th percentile) of −7 days (−21 to 0 days)). Moreover, echocardiography was performed at the time of the annual transthoracic echocardiographic examination instead of at the time of recruitment or taking NT-proBNP measurements. Fifth, natriuretic peptide levels are influenced by various factors, such as age and BMI. These confounding factors may have affected the association between GNRI, SMI, and natriuretic peptide levels. Sixth, we did not investigate the association between post-dialysis fatigue and natriuretic peptide. Therefore, future studies are warranted to address these issues.

## 5. Conclusions

Most patients with higher NT-proBNP levels had muscle attenuation and/or inflammation and body fluid overload. Elevated NT-proBNP levels due to such an imbalance in the body fluid composition are associated with a low health-related QOL. In our daily clinical practice, we recommend resetting the dry weight when the NT-proBNP levels increase in patients undergoing hemodialysis. It is also important to assess their nutritional status, QOL, and new-onset cardiovascular disease.

## Figures and Tables

**Table 1:** Population characteristics according to the pre-hemodialysis NT-proBNP quartiles.

Patients’ Characteristics	NT-proBNP, pg/mL				*p*
	Quartile 1 172–1700 (n = 80)	Quartile 2 1720–3410 (n = 81)	Quartile 3 3430–7170 (n = 81)	Quartile 4 7200–69,000 (n = 80)	
Age, years	62 (49–71)	67 (54–74)	68 (57–76)	70 (63–75)	<0.001
Male sex, n (%)	63 (78.8)	52 (64.2)	49 (60.5)	63 (78.8)	0.76
Diabetes mellitus, n (%)	45 (56.3)	46 (56.8)	50 (61.7)	42 (52.5)	0.81
Dialysis vintage, months	48 (23–98)	81 (38–126)	67 (35–128)	88 (45–155)	0.001
Cardiovascular disease, n (%)	13 (16.3)	15 (18.5)	10 (12.4)	17 (21.3)	0.48
Smoker, n (%)	17 (21.3)	27 (33.3)	23 (28.4)	19 (23.8)	0.76
Body mass index, kg/m^2^	24.3 (20.8–27.3)	22.9 (20.4–25.1)	21.1 (19.4–24.3)	21.0 (19.2–23.9)	<0.001
Pre-dialysis body weight, kg	69.3 (58.3–82.2)	62.6 (53.0–72.8)	60.3 (51.2–67.8)	60.2 (52.8–68.3)	<0.001
Post-dialysis body weight, kg	65.9 (56.3–79.0)	59.9 (50.6–69.2)	56.5 (48.4–64.1)	56.7 (49.8–65.1)	<0.001
Ultrafiltration volume, L	3.2 (2.5–4.3)	2.9 (2.4–4.0)	2.9 (2.2–4.0)	2.9 (2.3–3.7)	0.06
Ultrafiltration volume, % of body weight	4.9 (4.0–5.9)	5.2 (4.4–6.0)	5.2 (4.1–6.2)	5.1 (4.1–6.2)	0.31
Pre-dialysis systolic BP, mmHg	140 (126–154)	144 (129–159)	150 (131–165)	154 (133–166)	0.001
Post-dialysis systolic BP, mmHg	128 (115–148)	133 (119–150)	145 (133–165)	144 (125–164)	<0.001
Pre-dialysis diastolic BP, mmHg	79 (68–88)	74 (68–88)	78 (68–87)	76 (68–85)	0.98
Post-dialysis diastolic BP, mmHg	75 (69–86)	75 (66–84)	78 (66–90)	75 (65–86)	0.95
Total protein, g/dL	6.7 (6.4–6.9)	6.5 (6.2–6.7)	6.5 (6.2–6.9)	6.5 (6.2–6.7)	0.14
Serum albumin, mg/dL	3.7 (3.5–3.8)	3.6 (3.5–3.8)	3.5 (3.4–3.7)	3.5 (3.3–3.7)	<0.001
Blood urea nitrogen, mg/dL	59 (50–71)	61 (53–72)	58 (49–67)	53 (45–62)	0.001
Serum creatinine, mg/dL	11.36 (9.41–12.87)	10.67 (9.38–11.89)	9.93 (8.69–11.60)	9.56 (8.45–10.79)	<0.001
Serum sodium, mEq/L	139 (138–143)	139 (138–141)	139 (137–141)	139 (138–140)	0.037
Serum potassium, mEq/L	4.7 (4.2–5.0)	5.0 (4.5–5.6)	4.8 (4.5–5.3)	4.8 (4.4–5.2)	0.24
Serum chloride, mEq/L	103 (100–105)	103 (102–105)	104 (102–106)	104 (101–106)	0.60
Serum calcium, mg/dL	8.7 (8.3–8.9)	8.7 (8.3–9.0)	8.5 (8.2–8.9)	8.7 (8.2–8.9)	0.78
Serum phosphorus, mg/dL	5.6 (5.0–6.2)	5.6 (5.2–6.4)	5.5 (5.0–6.4)	5.5 (4.8–6.1)	0.31
Uric acid, mg/dL	8.1 (7.3–9.2)	7.8 (7.1–8.5)	7.6 (6.9–8.3)	7.3 (6.5–8.2)	<0.001
Total cholesterol, mg/dL	169 (151–198)	169 (142–205)	159 (140–188)	153 (133–177)	0.010
Triglyceride, mg/dL	138 (81–204)	104 (67–154)	91 (65–133)	95 (62–114)	<0.001
Blood glucose, mg/dL	117 (98–158)	109 (89–158)	111 (94–146)	112 (96–139)	0.73
Fe, μg/dL	62 (50–82)	71 (56–84)	65 (54–82)	65 (51–81)	0.43
TIBC, μg/dL	262 (233–314)	253 (222–277)	260 (234–286)	267 (242–302)	0.31
Ferritin, ng/mL	49 (29–98)	66 (40–97)	54 (33–92)	59 (33–101)	0.70
Hemoglobin, g/dL	11.4 (10.7–12.2)	11.2 (10.8–11.9)	11.1 (10.7–11.8)	11.0 (10.5–11.6)	<0.001
Hematocrit, %	34.7 (32.6–37.5)	33.9 (32.4–36.1)	33.8 (32.8–35.5)	33.4 (31.4–35.9)	0.010
C-reactive protein, mg/dL	0.14 (0.05–0.22)	0.07 (0.04–0.21)	0.08 (0.04–0.26)	0.14 (0.06–0.38)	<0.001
Kt/Vurea	1.71 (1.54–1.94)	1.88 (1.67–2.08)	1.87 (1.68–2.18)	1.85 (1.71–2.07)	0.07
Geriatric nutritional risk index	101 (95–108)	97 (92–102)	93 (88–101)	93 (87–98)	0.003
CTR in men (n = 227), %	50.2 (46.4–52.9)	52.6 (50.8–53.9)	51.7 (49.4–51.7)	56.0 (52.6–59.3)	<0.001
CTR in women (n = 95), %	48.4 (46.0–52.3)	49.4 (45.1–52.2)	50.2 (46.7–53.5)	52.8 (49.4–55.2)	<0.001

NT-proBNP, pre-dialysis N-terminal pro-B-type natriuretic peptide; BP, pre- and post-dialysis blood pressure; Kt/V, single-pool Kt/Vurea; CTR, the cardiothoracic index.

**Table 2:** Factors independently associated with log_10_-transformed NT-proBNP.

Variables	Unadjusted		Model 1		Model 2	
	β (95% CI)	*p*	β (95% CI)	*p*	β (95% CI)	*p*
Age	0.29 (0.007–0.016)	<0.001			0.03 (−0.004–0.006)	0.59
Dialysis vintage, months	0.18 (0.000–0.002)	0.001	0.08 (−0.000–0.001)	0.08	0.05 (−0.000–0.001)	0.36
Body mass index, kg/m^2^	−0.22 (−0.042–−0.014)	<0.001			−0.01 (−0.013–0.016)	0.86
Post-dialysis systolic BP, mmHg	0.25 (0.003–0.076)	<0.001	0.19 (0.002–0.006)	0.006	0.15 (0.001–0.005)	0.006
Serum albumin, mg/dL	−0.26 (−0.630–−0.262)	<0.001			−0.08 (−0.325–0.059)	0.18
Hemoglobin, g/dL	−0.18 (−0.141–−0.037)	<0.001	−0.09 (−0.097–0.010)	0.11	−0.02 (−0.060–0.041)	0.11
C-reactive protein, mg/dL	0.22 (0.083–0.243)	<0.001	0.16 (0.044–0.201)	0.002	0.13 (0.024–0.178)	0.002
Geriatric nutritional risk index *	−0.29 (−0.022–−0.010)	<0.001	−0.20 (−0.016–−0.004)	<0.001		
Phase angle, °	−0.45 (−0.253–−0.162)	<0.001			−0.34 (−0.218–−0.091)	<0.001

BP, pre- and post-dialysis blood pressure; NT-proBNP, pre-dialysis N-terminal pro-B-type natriuretic peptide. Model 1, adjusted for dialysis vintage, post-dialysis systolic BP, hemoglobin, C-reactive protein, and geriatric nutritional risk index; model 2, adjusted for age, dialysis vintage, post-dialysis systolic BP, serum albumin, hemoglobin, C-reactive protein, hemoglobin, and PhA. * If age was used as the dependent variable, the significance difference remained similar. If geriatric nutritional risk index instead of age, body mass index, and serum albumin was used as dependent variables, post-dialysis systolic BP and C-reactive protein remained as independent associated factors.

**Table 3:** Echocardiographic findings according to the pre-hemodialysis NT-proBNP quartiles.

Patients’ Characteristics	NT-proBNP, pg/mL				*p*
	Quartile 1 172–1700 (n = 80)	Quartile 2 1720–3410 (n = 81)	Quartile 3 3430–7170 (n = 81)	Quartile 4 7200–69,000 (n = 80)	
LAD, mm	36.0 (32.0–38.6)	36.2 (33.3–39.2)	37.0 (33.0–41.5)	40.2 (36.1–44.4)	<0.001
LVDd, mm	45.4 (39.9–49.3)	45.0 (41.5–51.7)	44.3 (40.4–49.3)	48.0 (42.0–51.4)	0.046
LVDs, mm	29.4 (25.0–32.1)	29.8 (24.5–34.2)	28.8 (26.5–33.0)	31.3 (27.8–35.3)	0.003
PWT, mm	10.1 (9.0–11.7)	10.5 (9.6–12.0)	10.8 (9.8–12.8)	11.6 (10.0–12.9)	<0.001
IVST, mm	10.2 (8.6–12.0)	11.2 (10.0–12.3)	11.0 (10.0–12.2)	11.8 (10.0–13.2)	<0.001
EF	65.3 (60.4–70.0)	65.1 (59.6–72.6)	63.3 (58.9–68.1)	62.1 (56.1–67.8)	<0.001
LVMI, g/m^2^	87 (72–106)	107 (93–134)	107 (89–126)	124 (104–149)	<0.001

LAD, left atrial dimension; LVDd, left ventricular end-diastolic diameter; LVDs, left ventricular end-systolic diameter; PWT, left ventricular posterior wall thickness; IVST, interventricular septum thickness; EF, ejection fraction; LVMI, left ventricular mass index.

**Table 4:** Quality-of-life domains according to the pre-hemodialysis NT-proBNP quartiles.

QoL Domains	NT-proBNP, pg/mL				*p*
	Quartile 1 172–1700 (n = 80)	Quartile 2 1720–3410 (n = 81)	Quartile 3 3430–7170 (n = 81)	Quartile 4 7200–69,000 (n = 80)	
*Physical health domains*					
Physical functioning	85 (66–95)	85 (70–95)	75 (60–90)	70 (50–80)	<0.001
Role limitations caused by physical health problems	75 (25–100)	75 (13–100)	75 (0–100)	38 (0–100)	0.004
Bodily pain	78 (55–90)	70 (45–90)	68 (45–90)	59 (45–80)	0.013
General health	50 (40–55)	50 (35–60)	45 (35–50)	45 (30–50)	0.05
*Mental health domains*					
Vitality	58 (40–70)	60 (45–75)	55 (40–75)	50 (31–65)	0.025
Social functioning	88 (50–100)	75 (50–100)	75 (56–100)	75 (50–100)	0.14
Role limitations caused by emotional health problems	100 (83–100)	100 (33–100)	67 (0–100)	67 (0–100)	0.008
Emotional well-being	72 (56–84)	76 (56–88)	68 (56–82)	64 (48–80)	0.018
*Kidney disease-specific domains*					
Symptoms	83 (73–90)	88 (78–94)	83 (74–95)	79 (71–88)	0.031
Effects of kidney disease	78 (69–88)	81 (69–91)	78 (63–91)	77 (63–88)	0.10
Burden of kidney disease	38 (25–50)	44 (25–56)	38 (25–56)	31 (25–50)	0.83
Work status	50 (0–100)	50 (0–100)	50 (0–50)	50 (0–50)	0.003
Cognitive function	93 (87–100)	93 (87–100)	93 (80–100)	93 (80–100)	0.57
Quality of social interaction	87 (67–100)	93 (50–100)	93 (55–100)	68 (47–87)	0.94
Sleep	55 (43–70)	68 (50–80)	63 (53–80)	59 (48–73)	0.32
Social support	67 (67–83)	67 (67–83)	67 (67–100)	67 (67–83)	0.36
Dialysis staff encouragement	75 (50–100)	75 (50–100)	75 (67–100)	75 (67–88)	0.38
Patient satisfaction	83 (67–100)	83 (83–100)	83 (67–100)	83 (67–83)	0.60

NT-proBNP, pre-dialysis N-terminal pro-B-type natriuretic peptide.

## Data Availability

The datasets generated and analyzed during the current study are available from the corresponding author on reasonable request.

## Supplementary Materials

The following supporting information can be downloaded at: https://www.mdpi.com/article/10.3390/jcm12237356/s1. Table S1: Body fluid composition in men according to the pre-hemodialysis NT-proBNP quartiles; Table S2: Body fluid composition in women according to the pre-hemodialysis NT-proBNP quartiles; Table S3: NT-proBNP as an independent factor associated with quality-of-life domains.
