# Supplementary material for: Associations between N-Terminal Pro-B-Type Natriuretic Peptide, Body Fluid Imbalance and Quality of Life in Patients Undergoing Hemodialysis: A Cross-Sectional Study
Source: J Clin Med. 2023 Nov 28;12(23):7356. doi: 10.3390/jcm12237356 (PMC10706951; doi:10.3390/jcm12237356)
Supplement: Supplementary file 1 [file jcm-12-07356-s001.zip › YK_finalized_jcm-2651166_Supplemental table1.pdf]

Supplemental Table 1. Body fluid composition in men according to pre-hemodialysis NT-proBNP quartiles

Body fluid composition	NT-proBNP, pg/mL Quartile 1 172–1700 (n = 63)	Quartile 2 1720–3,410 (n = 52)	Quartile 3 3430–7,170 (n = 49)	Quartile 4 7200–69,000 (n = 63)	*P*
Body weight, kg	69.2 (58.0–80.0)	65.7 (57.4–71.1)	62.0 (55.7–72.6)	58.6 (52.0–66.0)	<0.001
Body surface area, m^2	1.79 (1.67–1.97)	1.75 (1.65–1.84)	1.69 (1.59–1.83)	1.66 (1.56–1.77)	<0.001
Total body water, l	36.9 (33.4–42.8)	35.9 (33.7–40.4)	34.4 (31.5–40.4)	33.7 (31.2–37.2)	0.002
Total body water, l per BSA	21.0 (19.5–22.4)	21.1 (19.8–21.9)	20.6 (19.3–22.0)	20.3 (19.4–21.8)	0.41
Intracellular water, l	23.4 (20.3–26.9)	22.1 (20.9–25.1)	21.3 (18.8–22.9)	20.4 (18.7–22.9)	<0.001
Intracellular water, l per BSA	13.0 (12.2–14.0)	13.0 (12.3–13.4)	12.5 (11.8–12.5)	12.2 (11.6–13.3)	0.014
Extracellular water, l	14.3 (12.9–16.5)	13.9 (12.8–15.3)	13.3 (12.3–15.5)	13.5 (12.5–14.7)	0.10
Extracellular water, l per BSA	8.0 (7.4–8.5)	8.1 (7.6–8.5)	7.0 (7.4–8.6)	8.0 (7.7–8.5)	0.041
Extracellular water to Intracellular water ratio	0.64 (0.61–0.65)	0.63 (0.62–0.66)	0.65 (0.64–0.67)	0.67 (0.66–0.69)	0.003
Protein, kg	10.1 (8.8–11.6)	9.5 (9.0–10.9)	9.2 (8.1–10.7)	8.8 (8.0–9.9)	<0.001
%Protein, %	14.5 (13.6–15.8)	15.1 (13.8–15.8)	14.8 (13.6–16.2)	14.9 (13.9–16.1)	0.09
Mineral, kg	3.38 (2.92–3.93)	3.29 (3.02–3.66)	3.27 (2.77–3.65)	3.04 (2.81–3.43)	0.003
%Mineral, %	5.0 (4.6–5.4)	5.0 (4.7–5.5)	4.9 (4.5–5.8)	5.2 (4.8–5.6)	0.007
Fat, kg	18.5 (12.0–23.3)	16.0 (11.1–21.0)	14.4 (10.2–21.0)	12.8 (9.4–17.7)	<0.001
%Fat, %	26.7 (19.1–30.8)	23.5 (18.6–29.7)	24.3 (16.8–30.7)	22.7 (16.8–28.2)	0.004
Free fat mass, kg	50.5 (45.0–58.5)	48.8 (45.9–54.8)	46.4 (42.5–54.6)	45.6 (42.1–50.7)	0.005

Free fat mass, %	73.2 (69.2–80.9)	76.5 (70.4–81.4)	75.7 (69.3–83.2)	77.3 (71.8–83.2)	0.16
Body cell mass, kg	32.4 (29.1–38.6)	31.8 (30.0–35.8)	30.5 (27.0–35.6)	29.6 (26.6–33.1)	0.006
Body cell mass, %	48.4 (45.9–52.2)	50.3 (45.9–52.5)	48.7 (44.9–54.4)	49.7 (46.0–53.6)	0.82
Phase angle	6.1 (5.3–6.9)	5.8 (5.0–6.4)	5.3 (4.5–6.1)	4.7 (4.0–5.4)	<0.001
Skeletal muscle mass index, kg/m^2	7.6 (7.2–8.7)	7.6 (7.1–8.3)	7.4 (6.7–8.3)	7.2 (6.3–8.0)	0.003
