# Supplementary material for: Associations between N-Terminal Pro-B-Type Natriuretic Peptide, Body Fluid Imbalance and Quality of Life in Patients Undergoing Hemodialysis: A Cross-Sectional Study
Source: J Clin Med. 2023 Nov 28;12(23):7356. doi: 10.3390/jcm12237356 (PMC10706951; doi:10.3390/jcm12237356)
Supplement: Supplementary file 1 [file jcm-12-07356-s001.zip › YK_finalized_jcm-2651166_Supplemental table2.pdf]

Supplemental Table 2. Body fluid composition in women according to the pre-hemodialysis NT-proBNP quartiles

Body fluid composition	NT-proBNP, pg/mL				P
	Quartile 1 172–1700 (n = 17)	Quartile 2 1720–3410 (n = 29)	Quartile 3 3430–7170 (n = 32)	Quartile 4 7200–69,000 (n = 17)	
Body weight, kg	58.1 (47.1–64.8)	48.4 (43.7–54.3)	48.1 (43.6–54.6)	46.7 (41.0–55.3)	0.39
Body surface area, m^2	1.58 (1.42–1.63)	1.43 (1.36–1.48)	1.45 (1.35–1.54)	1.42 (1.31–1.51)	0.53
Total body water, l	27.1 (24.6–29.2)	25.1 (23.7–26.9)	26.1 (23.4–28.5)	24.3 (22.2–27.1)	0.48
Total body water, l per BSA	17.6 (16.6–18.8)	17.9 (17.2–18.5)	18.2 (17.0–19.0)	17.4 (15.9–18.8)	0.77
Intracellular water, l	16.5 (15.1–18.1)	15.3 (14.3–16.6)	15.7 (14.3–17.5)	14.4 (13.1–16.6)	0.29
Intracellular water, l per BSA	10.6 (10.2–11.6)	11.0 (10.3–11.3)	11.0 (10.4–11.6)	10.5 (9.5–11.3)	0.33
Extracellular water, l	10.6 (9.4–11.4)	9.8 (9.4–10.5)	10.5 (9.3–11.3)	9.9 (9.0–10.7)	0.93
Extracellular water, l per BSA	6.8 (6.5–7.1)	7.0 (6.6–7.3)	7.2 (6.7–7.5)	6.9 (6.4–7.6)	0.43
Extracellular water to Intracellular water ratio	0.64 (0.61–0.65)	0.63 (0.62–0.66)	0.65 (0.64–0.67)	0.67 (0.66–0.69)	0.003
Protein, kg	7.1 (6.5–7.8)	6.6 (6.2–7.2)	6.8 (6.2–7.5)	6.2 (5.7–7.2)	0.27
%Protein, %	12.2 (11.7–13.8)	13.6 (12.2–14.9)	14.2 (12.6–15.6)	13.7 (11.9–15.2)	0.71
Mineral, kg	2.54 (2.23–2.76)	2.50 (2.29–2.62)	2.59 (2.38–2.79)	2.40 (2.27–2.65)	0.71
%Mineral, %	4.5 (4.1–5.2)	5.0 (4.5–5.6)	5.4 (4.9–6.0)	5.3 (4.3–6.2)	0.09
Fat, kg	21.2 (14.2–26.1)	14.7 (10.1–19.7)	11.8 (9.2–17.8)	12.9 (7.9–21.0)	0.50
%Fat, %	36.9 (28.2–40.1)	28.9 (22.7–37.5)	25.4 (20.1–34.8)	28.4 (18.5–38.3)	0.39
Free fat mass, kg	36.9 (33.4–39.6)	34.0 (32.1–36.9)	35.4 (31.8–38.6)	33.2 (30.1–36.9)	0.13

Free fat mass, %	63.1 (60.0–71.8)	71.1 (62.5–77.3)	74.6 (65.2–79.9)	71.6 (61.7–81.5)	0.034
Body cell mass, kg	24.0 (21.1–26.4)	21.9 (20.4–23.4)	22.3 (20.7–24.4)	20.1 (18.3–21.6)	0.014
Body cell mass, %	41.3 (39.1–45.5)	46.2 (40.7–49.7)	48.4 (44.0–51.4)	46.5 (38.5–49.9)	0.09
Phase angle	5.1 (4.8–6.2)	5.1 (4.6–5.4)	4.8 (4.3–5.1)	4.0 (3.4–4.3)	<0.001
Skeletal muscle mass index, kg/m^2	6.4 (5.5–7.4)	6.0 (5.5–6.4)	6.0 (5.5–6.6)	5.3 (4.9–6.2)	0.048
