# Supplementary material for: Associations between N-Terminal Pro-B-Type Natriuretic Peptide, Body Fluid Imbalance and Quality of Life in Patients Undergoing Hemodialysis: A Cross-Sectional Study
Source: J Clin Med. 2023 Nov 28;12(23):7356. doi: 10.3390/jcm12237356 (PMC10706951; doi:10.3390/jcm12237356)
Supplement: Supplementary file 1 [file jcm-12-07356-s001.zip › YK_finalized_jcm-2651166_Supplemental table3.pdf]

Supplemental Table 3. NT-proBNP as an independent factor associated with quality of life domains

QoL domains	Unstandardized B (95% CI)	Standardized β	*P*
	Physical health domains		
Physical functioning			
Unadjusted	−10.08 (−15.19, −4.96)	−0.21	<0.001
Multivariable-adjusted[1]	−8.78 (−14.23, −3.32)	−0.18	0.002
Role limitations caused by physical health problems			
Unadjusted	−14.03 (−23.41, −4.64)	−0.16	0.004
Multivariable-adjusted[2]	−10.59 (−20.56, −0.62)	−0.12	0.037
Bodily pain			
Unadjusted	−7.46 (−13.33, −1.58)	−0.14	0.013
Multivariable-adjusted[3]	−6.42 (−12.37, −0.48)	−0.12	0.034
General health			
Unadjusted	−3.82 (−7.67, 0.04)	−0.11	0.05
Multivariable-adjusted[4]	−5.43 (−9.42, −1.45)	−0.15	0.008
	Mental health domains		
Vitality			
Unadjusted	−5.79 (−10.87, −0.71)	−0.12	0.026
Multivariable-adjusted[5]	−4.93 (−10.24, 0.39)	−0.11	0.07
Social functioning			
Unadjusted	−4.68 (−10.94, 1.57)	−0.08	0.14
Multivariable-adjusted[6]	−3.97 (−10.45, 2.51)	−0.07	0.07
**Role limitations caused by emotional health problem**			
Unadjusted	−13.17 (−22.86, −3.48)	−0.15	0.008
Multivariable-adjusted[7]	−10.60 (−20.70, −0.49)	−0.12	0.040
Emotional well-being			
Unadjusted	−6.05 (−11.04, −1.06)	−0.13	0.018
Multivariable-adjusted[8]	−6.05 (−11.04, −1.06)	−0.13	0.018
	Kidney disease-specific domains		
Symptoms			

Unadjusted	−4.98 (−9.52, −0.45)	−0.12	0.031
Multivariable-adjusted[9]	−5.11 (−9.61, −0.62)	−0.12	0.026
Effects of kidney disease			
Unadjusted	−4.07 (−8.87, 0.73)	−0.09	0.10
Multivariable-adjusted[10]	−2.87 (−7.82, 2.08)	−0.07	0.26
Burden of kidney disease			
Unadjusted	−0.53 (−5.49, 4.42)	−0.01	0.83
Multivariable-adjusted[11]	−1.13 (−6.16, 3.90)	−0.03	0.66
Work status			
Unadjusted	−13.24 (−21.82, −4.66)	−0.17	0.003
Multivariable-adjusted[12]	−10.02 (−18.87, −1.17)	−0.17	0.027
Cognitive function*			
Unadjusted	−1.41 (−6.32, 3.49)	−0.03	0.57
Quality of social interaction			
Unadjusted	−0.20 (−5.28, 4.87)	−0.004	0.94
Multivariable-adjusted[13]	−2.87 (−7.82, 2.08)	−0.07	0.26
Sleep			
Unadjusted	2.40 (−2.29, 7.09)	0.06	0.32
Multivariable-adjusted[14]	1.06 (−3.78, 5.90)	0.02	0.67
Social support*			
Unadjusted	−2.56 (−8.09, 2.97)	−0.05	0.36
Dialysis staff encouragement			
Unadjusted	−2.53 (−8.20, 3.14)	−0.05	0.38
Multivariable-adjusted[15]	−1.28 (−7.01, 4.44)	−0.02	0.66
Patient satisfaction*			
Unadjusted	−1.25 (−6.05, 3.55)	−0.03	0.61

1, adjusted for geriatric nutritional risk index (GNRI); 2, C-reactive protein and GNRI; 3, dialysis vintage; 4, age; 5, GNRI; 6, serum albumin; 7, GNRI; 8, no other variables; 9, gender and diabetes mellitus; 10, serum albumin; 11, gender, diabetes mellitus, and C-reactive protein; 12, diabetes mellitus and GNRI; 13, age; 14, age, gender, and diabetes mellitus; 15, dialysis vintage
*No explanatory variables that showed a significant relationship ($P < 0.05$) with these QOL domains
